# Early Detection of Fluid Retention in Patients with Advanced Heart Failure: A Review of a Novel Multisensory Algorithm, HeartLogic^TM^

**DOI:** 10.3390/s21041361

**Published:** 2021-02-15

**Authors:** Michelle Feijen, Anastasia D. Egorova, Saskia L. M. A. Beeres, Roderick W. Treskes

**Affiliations:** Department of Cardiology, Leiden University Medical Center, Albinusdreef 2, 2333 ZA Leiden, The Netherlands; m.feijen@lumc.nl (M.F.); a.egorova@lumc.nl (A.D.E.); r.w.treskes@lumc.nl (R.W.T.)

**Keywords:** heart failure, multi sensor remote monitoring, HeartLogic^TM^, cardiac implantable electronic devices, fluid retention, admissions

## Abstract

Heart failure (HF) hospitalisations due to decompensation are associated with shorter life expectancy and lower quality of life. These hospitalisations pose a significant burden on the patients, doctors and healthcare resources. Early detection of an upcoming episode of decompensation may facilitate timely optimisation of the ambulatory medical treatment and thereby prevent heart-failure-related hospitalisations. The HeartLogic^TM^ algorithm combines data from five sensors of cardiac implantable electronic devices into a cumulative index value. It has been developed for early detection of fluid retention in heart failure patients. This review aims to provide an overview of the current literature and experience with the HeartLogic^TM^ algorithm, illustrate how the index can be implemented in daily clinical practice and discuss ongoing studies and potential future developments of interest.

## 1. Introduction

Heart failure is a syndrome that is defined by the European Society of Cardiology as “a clinical syndrome characterized by typical symptoms (e.g., breathlessness, ankle swelling and fatigue) that may be accompanied by signs (e.g., elevated jugular venous pressure, pulmonary crackles and peripheral edema) caused by a structural and/or functional cardiac abnormality, resulting in a reduced cardiac output and/or elevated intracardiac pressures at rest or during stress” [1]. It results in substantial mortality, morbidity and worsening of clinical status, often leading to hospitalisations because of fluid retention [2,3,4]. These hospitalisations impair quality of life, are a marker of poor prognosis and pose a significant burden on healthcare resources [3,4]. Advances in pharmacological therapy, revascularisation techniques and cardiac implantable electronic devices (CIEDs) have significantly improved quality of life and survival of patients with heart failure over the past decades [5,6,7,8,9,10]. Nevertheless, industrialised countries still spend 1–2% of their healthcare budgets on heart failure patients. Most of these costs (estimated 80%) are attributable to hospitalisations for decompensated heart failure [11]. It is expected that these hospitalisation associated costs will further rise in the near future as the prevalence of heart failure increases with the aging population and improvements in treatment [12].

The mainstay of preventing heart failure admissions is early detection of an episode of upcoming decompensation. Timely recognition, however, remains challenging as in daily practice worsening symptoms occur relatively late [13]. Recently, various tele- and remote monitoring initiatives have gained interest for their promise in detecting an upcoming episode of decompensation before patients experience clinical symptoms. This may facilitate timely adjustment of ambulatory medical treatment and prevent heart-failure-related hospitalisations [14]. Most telemonitoring initiatives are focussed on measuring body weight and blood pressure at home. Several meta-analyses suggested clinical benefits, but the results of numerous prospective clinical studies could not confirm this [14,15,16,17,18,19,20,21,22,23,24,25,26].

Another management strategy is remote monitoring through implantable hemodynamic monitors. For example, the CardioMEMS^TM^ sensor is a permanent pulmonary artery wireless microelectromechanical sensor that enables daily measurement of filling pressures, which have been shown to increase several weeks before a period of decompensation becomes clinically apparent [26]. In the CHAMPION trial in the United States, there was a 37% reduction in heart failure hospitalisations in the CardioMEMS^TM^ group as compared to the standard care group [15]. Several studies assessing the utility of the CardioMEMS^TM^ sensor in broader cohorts of heart failure patients and in different healthcare settings are ongoing, including the MONITOR-HF study that investigates the efficacy and cost-effectiveness of hemodynamic pulmonary artery monitoring in addition to contemporary standard heart failure care in a high-quality western European healthcare system [27]. Despite the promising results of the current studies on pulmonary artery pressure monitoring, a disadvantage of this strategy is that this type of monitoring requires a dedicated invasive procedure to implant the CardioMEMS^TM^ sensor.

An alternative approach, taking advantage of the fact that many heart failure patients with a reduced left ventricular ejection fraction have a CIED, is telemonitoring through parameters that can be sensed by this device. Without additional effort of the patient, data can be continuously gathered and automatically transferred through the remote monitoring platform of the CIED. Earlier examples of these kinds of applications are OptiVol^TM^ and CorVue^TM^ that measured changes in the intrathoracic impedance. Studies evaluating intrathoracic impedance measurements revealed that this single marker is not robust enough in predicting an upcoming episode of decompensation [28,29,30,31]. More recently, efforts have been made to combine the data collected by multiple sensors to track physiological trends and combine them into one composite index. A promising example of this novel approach is the multisensor algorithm branded as HeartLogic^TM^ (Boston Scientific, Marlborough, MA, USA). This algorithm collects data regarding heart sounds, respiration, thoracic impedance, heart rate and activity data and combines them into an automatically calculated daily heart failure index. The United States Food and Drug Administration approved implantation of CIEDs with this feature in 2017. Since then, the number of patients in clinical practice with a CIED with the ability to measure the HeartLogic^TM^ index has been vastly increasing.

The aim of this review is to provide an overview of the current knowledge and literature on the HeartLogic^TM^ algorithm. Furthermore, three cases are presented to illustrate how the algorithm can be implemented in daily clinical practice, future studiesand potential developments of interest are discussed. In this paper, we demonstrate that the HeartLogic^TM^ algorithm is validated for early detection of fluid retention and highlight ongoing and future studies regarding the clinical implementation of the algorithm.

## 2. The HeartLogic^TM^ Algorithm and the Sensors behind It

**Figure 1:**
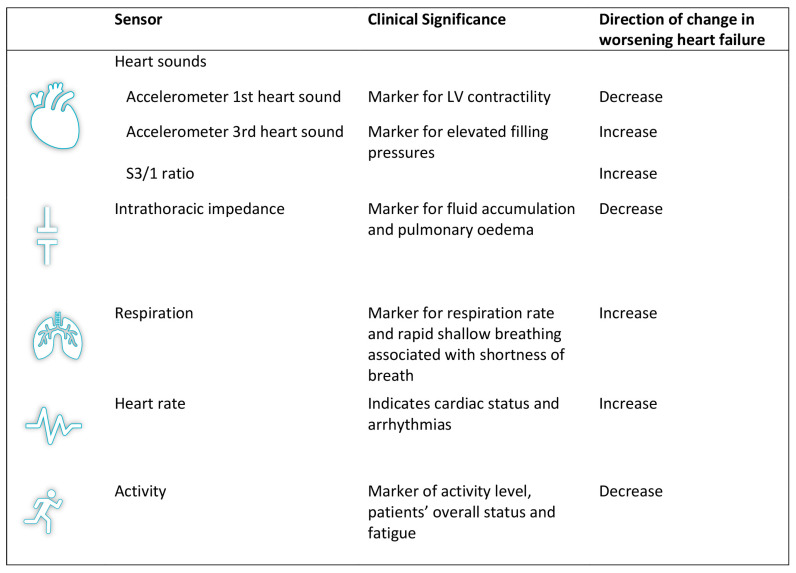
The sensors incorporated in the HeartLogic^TM^ index. S1, 1st heart sound; S3, 3rd heart sound; LV, left ventricle.

**Figure 2:**
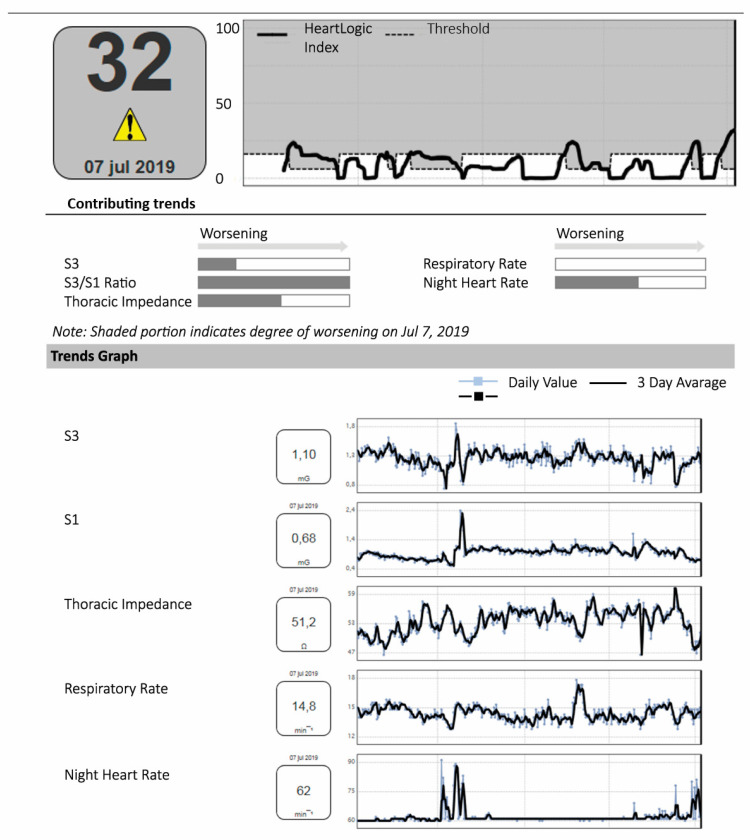
An example of a HeartLogic^TM^ report. The report states the cumulative HeartLogic^TM^ index (top left corner) and provides additional information on the data collected by the individual sensors (contributing trends and trend graph). S1, 1st heart sound; S3, 3rd heart sound.

The HeartLogic^TM^ algorithm is installed on implantable cardioverter defibrillator devices (ICDs) or cardiac resynchronisation therapy devices (CRTs) of the manufacturer: Boston Scientific. The following CIEDs (single and double chamber ICD, as well as CRT systems) are compatible with the HeartLogic^TM^ algorithm: PERCIVA^TM^, CHARISMA^TM^, MOMENTUM^TM^, RESONATE^TM^ and VIGILANT^TM^. The algorithm combines data obtained from five sensors (Figure 1) of the device into a single score, marketed as the HeartLogic^TM^ index [32]. Data from the following sensors are collected: first heart sound (S1), third heart sound (S3) and the derived S3/S1 ratio, thoracic impedance, respiration, night heart rate and patient activity. The first and third heart rate are derived from an accelerometer that is located in the CIED pulse generator. This accelerometer measures the movement of the right ventricular wall in diastole via the right ventricular lead. These movements follow a wave-type pattern. Typically, the amplitude of the wave (in milligravity) corresponds with the heart sounds heard at auscultation, which are registered by the device. S1, S3 and the ratio S3/S1 are derived this way [33]. Intrathoracic impedance is derived by the vector between the right ventricular lead and the device. As fluid conductivity is higher than that of air, intrathoracic impedance will decrease in case of intrathoracic fluid retention [34]. Respiratory rate and tidal volumes are derived from the intrathoracic impedance data [35]. Night heart rate is measured by calculating the RR interval between consecutive QRS complexes in the night. Patient activity is again measured by a device-based accelerometer that can convert specific motion characteristics into a score of activity. Data from these sensors are continuously collected by, and stored on, the CIED. The device then combines the input of these sensors (the exact formula of the algorithm is not released) to track physiological trends and provide the composite HeartLogic^TM^ index. This index, along with other device interrogation data, can then be transferred to the hospital via the manufacturer’s specific home monitoring system (Latitude, Boston Scientific). An example of a HeartLogic^TM^ report is provided in Figure 2.

After a CIED implantation, the algorithm is calibrated during a 30-day period. After this calibration period, deviations of the data provided by the five sensors are compared to the daily variations measured during the calibration period. Significant alternations in one or more of the sensors will result in a change of the composite index. Increased absolute value of the HeartLogic^TM^ index is associated with fluid accumulation. A low index is suggestive of a stable clinical status of the patient without signs of fluid retention. If the index surpasses the defined value of 16, a digital alert is given off in the home monitoring system and the medical team is alarmed. The threshold is dynamic and automatically lowers to 6 when an alert state (index of 16 or higher) is reached. The system will subsequently give weekly alerts until the index falls below 6.

There are a couple of features of the algorithm that need to be specifically mentioned: data are automatically transferred by the home monitoring system and no (auditory) alarms are given off to the patient directly. Patients need basic IT skills and a functional internet connection to transfer data via the home monitoring system. Data are reviewed at the hospital via a product specific platform by the dedicated heartcare professionals and alerts are not communicated by other means. Patients are therefore unaware of alerts until they are contacted by the healthcare professional. No additional hospital visits are required for data retrieval.

## 3. Evidence to Date

### 3.1. Literature Search and Selection

A structured review of the published studies on the HeartLogic^TM^ algorithm to date (31 December 2020) was performed. PubMed, Web of Science and Scopus were searched using the following terms: “heartlogic” and “heart failure”. The search was restricted to human studies in adults with heart failure and a CIED with the possibility to activate the HeartLogic^TM^ features. Editorials, case reports, reviews, book chapters, practice guidelines and abstract publications were excluded. The titles and abstracts of all studies retrieved from the literature search were screened. Studies, of which the relevance could not be ascertained based on the abstract, were screened in full text format. Extracted data included details of the study population, study design, statistical analysis, adjusted variables for multivariate analysis and duration of follow-up. Data on the primary and secondary endpoints regarding the performance of the alert (including sensitivity and specificity) and outcome measures (fluid retention/decompensated heart failure) were extracted.

**Figure 3:**
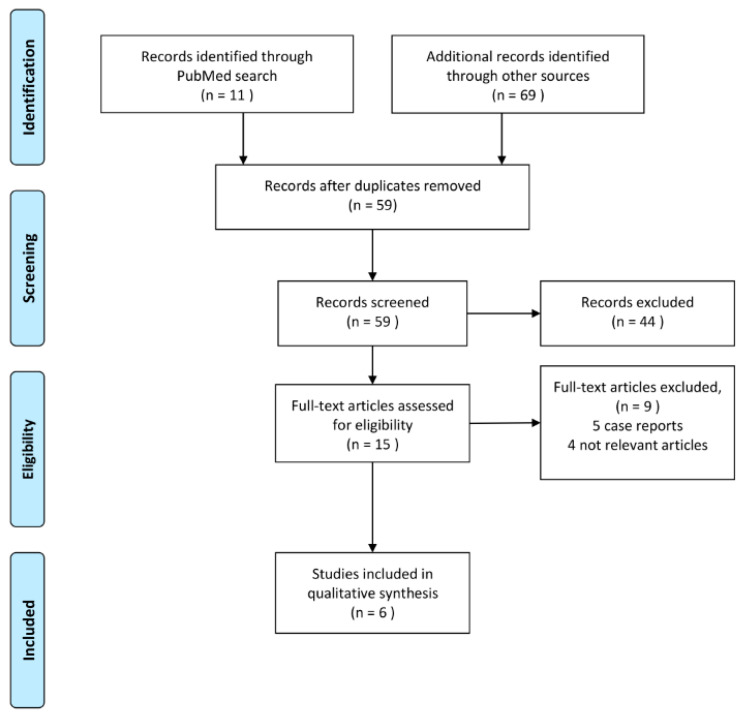
Overview of the literature search strategy and selection of relevant studies.

The above strategy resulted in 80 citations, from which 21 duplicates were removed. The remaining 59 citations were screened based on the title and abstract. Of these, 15 articles were additionally screened in full text format. In total, nine citations were excluded, five case reports and four articles that did not meet the inclusion criteria were deemed not relevant. Finally, six articles passed full text screening and were included in this review, Figure 3.

### 3.2. Studies Published to Date

Table 1 provides an overview of the publications that were obtained based on the above methodology. The results of these studies are analysed and discussed. 

Median age in all six studies was 60–71 years old and the majority of patients were male (73–81%), which is in line with other large heart failure studies [8,36,37]. In all six studies, patients had a severe to moderately reduced left ventricular ejection fraction (mean left ventricular ejection fraction (LVEF) 29–32%). The aetiology of heart failure was ischemic cardiomyopathy in 37–52% of patients. The remaining patients were classified in the heterogenous disease non-ischemic cardiomyopathy, e.g., dilated cardiomyopathy, toxic cardiomyopathy or hypertrophic cardiomyopathy. The majority of the patients were in the New York Heart Association functional class (NYHA) II–III (44–67% NYHA II, 18–51% NYHA III).

The MultiSENSE study was the landmark trial that hypothesised that the use of multiple sensors computed into the HeartLogic^TM^ algorithm would provide better insight into impeding decompensated heart failure episodes [32]. The study included 974 patients with heart failure who were followed for 12 months for heart failure events (defined as worsening of heart failure requiring clinical admission or need of intravenous medication). The study showed a sensitivity of 70% and specificity of 87.5% for heart failure events (heart failure admissions or unscheduled visits with intravenous treatment) and a clinically unexplained alert rate of 1.47 per patient year. The index had a negative predictive value of 99.8%, with a positive predictive value of 5.6%. This study showed that on average, an alert is given 34 days before a heart failure event (either hospitalisation of emergency care visit).

A post-hoc analysis of the MultiSENSE data by Gardner and colleagues showed that a HeartLogic^TM^ index of ≥16 compared with a HeartLogic^TM^ index <16 was associated with a 10.6-fold higher heart failure event ratio (0.80 events per patient year “in alert” state (HeartLogic^TM^ index ≥ 16) compared with 0.08 events per patient year “out of alert” state) [38]. Increased NT-Pro BNP (N-terminal B-type natriuretic peptide) levels (defined as > 1000 pg/mL) at baseline were also associated with a significant risk for heart failure events. Of interest, a high baseline NT-Pro BNP with an “out of alert” HeartLogic^TM^ index demonstrated only a modest event ratio of 0.16 events per patient year. In contrast, a low NT-Pro BNP and an “in alert” HeartLogic^TM^ index demonstrated a 3-fold higher event ratio of 0.47 events per patient year and a 23.5 times increased risk of a heart failure event compared to the “out of alert” and low NT-Pro BNP levels group. The group of patients who were “in alert” and had elevated NT-Pro BNP levels at baseline had a 50-fold higher event rate compared with patients “out of alert” state and a low NT-Pro BNP [38]. These data suggest that patients who are “out of alert” are indeed at a relatively low risk of heart failure events, even if they have a high baseline NT-Pro BNP. Additional subgroup analyses to evaluate the effects of demographics and comorbidities (amongst other atrial fibrillation and renal diseases) on the HeartLogic^TM^ index did not show significant interactions [38].

Capucci and colleagues performed a retrospective analysis of a case series of 67 patients (out of which, data of 58 patients were available) [33]. During a median follow-up of five months, 0.99 alerts per patient year were observed. Median early warning of a heart failure related event was 38 days in the case of hospitalisation and 12 days in the case of minor events of clinical deterioration of heart failure. An unexplained alert rate of 0.41 per patient year was observed (compared to the previously reported 1.47 in de MultiSENSE).

The results of two large prospective multicentre registries were published in 2020 [39,40]. Calò and colleagues aimed to evaluate the correlation between the S1 and S3 as measured by the CIED and echocardiographic indexes of systolic and diastolic left ventricular function [39]. CIED measured S3 was able to detect a restrictive filling pressure pattern (a surrogate for elevated left ventricular filling pressures), with 85% sensitivity and 82% specificity in 22 cases that were “in alert” [39]. CIED measured S1 was able to detect a poor LVEF (defined as EF < 35%) with a 28% sensitivity and a 88 % specificity in 22 cases [39].

In the second recently published prospective multicentre registry, Santini and colleagues reported 100 HeartLogic^TM^ alerts in 53 of the 104 patients (0.93 alerts per patient year) [40]. Sixty of these alerts (60%) were classified as clinically meaningful as they were associated with heart-failure-related events or resulted in active clinical actions (e.g., ambulant medication adjustments). In this study, 48 out of 60 alerts (80%) provided new relevant information to the treating team—i.e., the team was not aware of the signs and symptoms of fluid retention prior to the alert. These alerts triggered clinical actions in 43 out of 48 of the cases (90%), such as change in medication, outpatient clinic appointment or hospitalisation. An unexplained alert rate of 0.37 alerts per patient year was observed.

In the most recent study published in 2020, Mitter and colleagues performed a retrospective chart review of 38 New York patients with heart failure and active HeartLogic^TM^ features during the period of February–April 2020 [41]. No differences in HeartLogic^TM^ index before (1 February 2020–19 February 2020) or during (23 March 2020–15 April 2020) the first peak of the COVID-19 pandemic were observed. A significant drop in patient activity levels, with a corresponding drop in mean heart rate, a small increase in thoracic impedance and a less frequent S3 were observed during the first peak of the COVID-19 pandemic [41]. A decrease in the autonomic tone associated with reduced physical activity may have resulted in less congestion.

To date, studies focused primarily on the validation of the HeartLogic^TM^ algorithm and reported a sensitivity of 70% for detecting an upcoming heart failure episode [32]. On average, the patients spent 12–17% in an “in alert” state, triggered by a HeartLogic^TM^ index ≥ 16 [32,33,40]. The unexplained alert rate varied between 0.37 and 1.47 alerts per patient year in the various studies [32,33,40]. The HeartLogic^TM^ index alerts 34–38 days prior to a heart failure event in need of hospitalisation and 12 days before a minor clinical heart failure event [32,33]. A correlation between the CIED measured S3 and S1 and the echocardiographic surrogates of decompensation and progression of heart failure—a restrictive filling pattern and a poor left ventricular ejection fraction have been made [39]. These studies have validated the HeartLogic^TM^ index in predicting fluid retention and provided us with insights into the scope of functionalities of the algorithm. However, in order to further investigate the potential of the HeartLogic^TM^ index in modulating clinical outcomes—i.e., prevention of (re-)hospitalisation and shortening of admission duration, it is essential that this CIED modality be well embedded in a clinical care path.

## 4. HeartLogic in Daily Clinical Practice: Logistics and Course of Action

**Figure 4:**
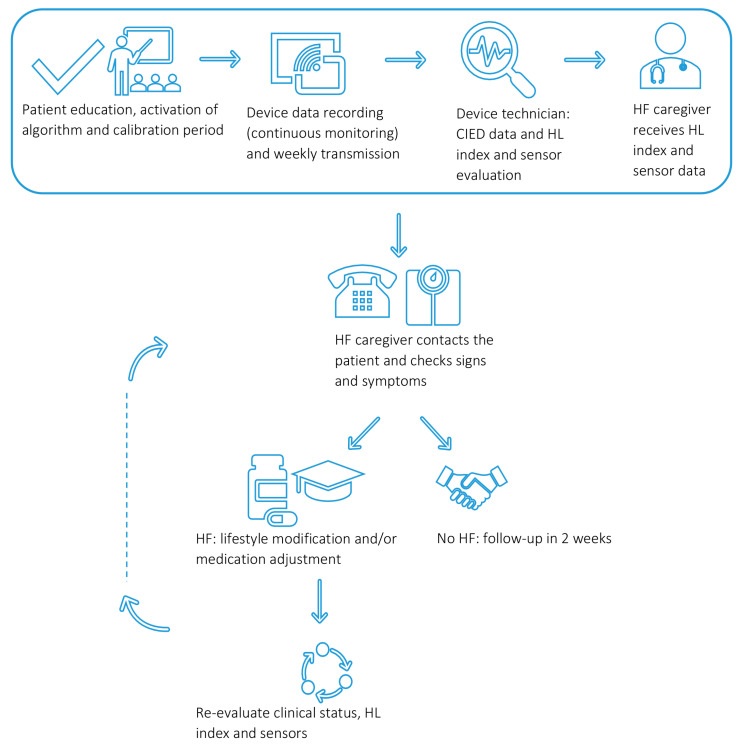
Schematic illustration of how the HeartLogic^TM^ index can be implemented in a clinical care path. HF, heart failure; HL, HeartLogic^TM^.

To explore the full range of clinical applications of the HeartLogic^TM^ algorithm, it is important for this CIED-based index to be incorporated into a clinical care path with defined roles and responsibilities of the healthcare professionals involved and an overall course of action in the case of an “in alert” patient status. Figure 4 provides a schematic example of how the HeartLogic^TM^ index can be embedded in clinical practice.

After CIED implantation or a pulse generator exchange procedure, the HeartLogic^TM^ features need to be activated by the device technician. After a CIED implantation, the Heartlogic^TM^ algorithm is first calibrated during a consecutive 30-day period. Following this calibration period, the deviations in the input provided by the sensors will be reflected in the value of the HeartLogic^TM^ index. The index value and the sensor input data will be monitored and stored in the CIED of the patient and transferred via the home monitoring system at regular intervals. These data can be accessed by the authorised healthcare providers using a secure web-based application. Data are typically initially reviewed by device technicians, who are supervised by a cardiologist-CIED specialist. In the case of a HeartLogic^TM^ alert, the device technician will analyse the CIED interrogation data and will inform the heart failure caregiver of the “in alert” status of the patient. The heart failure caregiver will contact the patient to check for potential signs and symptoms of fluid retention. In the case of (suspicion of) decompensated heart failure, this will be managed accordingly with lifestyle advice, medication adjustments and clinical evaluation as deemed necessary. The effect of the above intervention will be evaluated by reassessment of the clinical condition of the patient and the HeartLogic^TM^ index.

Three cases are discussed next to illustrate the implementation of the HeartLogic^TM^ index in our tertiary hospital.

### 4.1. Case 1: HeartLogic^TM^ Index as Part of a Heart Failure Care Path

A 69-year-old male patient had an anterior wall infarction in 1986, and an ICD for primary prevention was implanted in 2005. He developed progressive heart failure despite optimal medical treatment. Coronary artery bypass grafting, mitral valve plasty, tricuspid valve plasty and aortic valve replacement were performed in September 2017. A surgical left ventricular lead was placed, and the pulse generator was upgraded to a CRT-D.

After calibration, the HeartLogic^TM^ feature was available for clinical use from January 2018, Figure 5. At this moment the patient was in NYHA functional class II and euvolemic. Laboratory testing revealed an eGFR (estimated glomerular filtration rate) of 44 mL/min/1.73 m^2^ and a pro-BNP level of 6474 ng/L. The HeartLogic^TM^ index was 8, which is on the upper limit of normal (point A). On February the 5th, the HeartLogic^TM^ alert was issued as the index crossed the programmed threshold of 16 (point B). A review of the remote data showed that the index had gradually increased to 17 over the last 2 weeks. We contacted the patient by phone: he reported no symptoms or signs of fluid retention. Re-evaluation by phone was performed 2 weeks later (point C). Despite a further increase in the HeartLogic^TM^ index to 25, he still reported to be asymptomatic although he admitted to having a larger fluid and salt intake than advised. We recommended him to adjust his intake. Thereafter, the HeartLogic^TM^ index stabilised, but did not decrease. Accordingly, an outpatient visit was scheduled (point D). At the outpatient clinic, the patient initially denied having symptoms. However, despite cardiac rehabilitation, his exercise capacity decreased over the last weeks and he experienced mild shortness of breath during exercise. Physical examination showed elevated jugular venous pressure and mild ankle oedema. Laboratory testing revealed a decrease in renal function with an eGFR of 33 mL/min/1.73 m^2^ and the pro-BNP level had increased to 9650 ng/L. The HeartLogic^TM^ index was 22. Accordingly, we concluded that the patient had gradually developed signs of fluid retention and increased the dosage of bumetanide. After this, exercise capacity increased and he successfully completed the cardiac rehabilitation program. Simultaneously, the HeartLogic^TM^ index declined to 0 (point E). At a follow-up visit in April, the patient had no signs of fluid retention. Laboratory testing revealed that the eGFR improved to 42 mL/min/1.73 m^2^ and that the pro-BNP level declined to 4173 ng/L. Currently, two and a half years after surgery, the patient is in a clinically stable condition (NYHA functional class II) and no decompensated heart failure hospitalisations have occurred. This case illustrates how the HeartLogic^TM^ index can successfully be incorporated into a structured heart failure care path and shows that the index may be of value in guiding ambulant medication adjustments.

### 4.2. Case 2: HeartLogic^TM^ Index as an Accurate Reflection of the Fluid (and Not Arrythmia) Status

**Figure 6:**
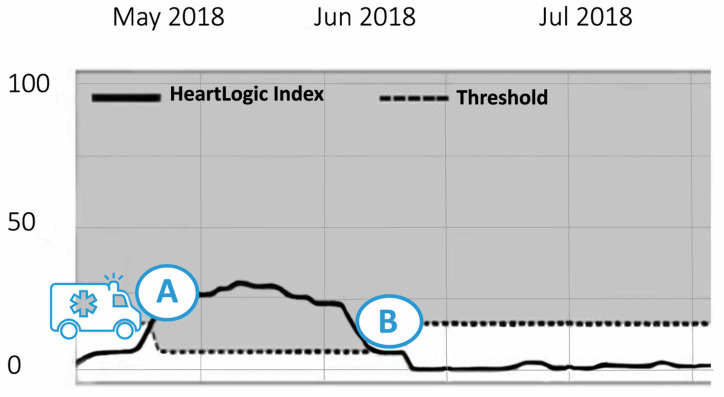
The course of HeartLogic^TM^ index of patient described in Case 2. Ambulance (19 April): Presentation with a ventricular tachycardia. A (29 May): Phone contact. HeartLogic^TM^ index exceeded the threshold of 16. B (12 June): Phone contact. HeartLogic^TM^ index 0.

A 49-year-old male patient with heart failure due to a familial hypertrophic cardiomyopathy had an ICD implanted for secondary prevention. In January 2018, his device was upgraded to a CRT-D because of progressive heart failure and a worsening LV function. Initially, his HeartLogic^TM^ index was well below the threshold of 16. In April 2018, he was admitted with dyspnoea based on a symptomatic slow ventricular tachycardia, which was terminated by antitachycardia pacing. Interestingly, his HeartLogic^TM^ index had not surpassed the threshold of 16, Figure 6. Indeed, clinical and echocardiographic evaluation revealed no signs of fluid retention or progression of heart failure as a trigger for this event. After adjusting antiarrhythmic medication and the tachycardia detection and antitachycardia pacing settings, he was discharged home.

On 29 May, 7 weeks after discharge, his HeartLogic^TM^ index exceeded the threshold. Remote interrogation of the CRT-D revealed no arrhythmias. The heart failure nurse contacted the patient by phone. The patient denied having symptoms of fluid retention although his exercise capacity was modest, and his weight had increased by two kilograms in four days. He said he had adhered to the lifestyle advice. The bumetanide dose was increased for three days, after which his weight returned to target and his exercise capacity improved. After two weeks, the HeartLogic^TM^ index decreased to 0. In this case, the HeartLogic^TM^ index accurately reflected the fluid status and symptoms of decompensation but did not give alerts in the case of arrhythmia that did not disturb the fluid status.

### 4.3. Case 3: HeartLogic^TM^ Index in a Complex Clinical Scenario of Decompensated Cardiorenal Failure

A 64-year-old female patient had heart failure due to an anthracycline medicated dilated cardiomyopathy. Despite optimal medical treatment and a mitral and tricuspid valve plasty in 2008, her left ventricular systolic function remained poor. Accordingly, an ICD for primary prevention was implanted in 2009. Since 2014, she had progressive heart failure and recurrent episodes of atrial fibrillation. Despite treatment with amiodarone, she developed permanent atrial fibrillation and dyspnoea NYHA class III. She was screened for a heart transplantation listing but was rejected due to stage 4 chronic kidney disease.

**Figure 7:**
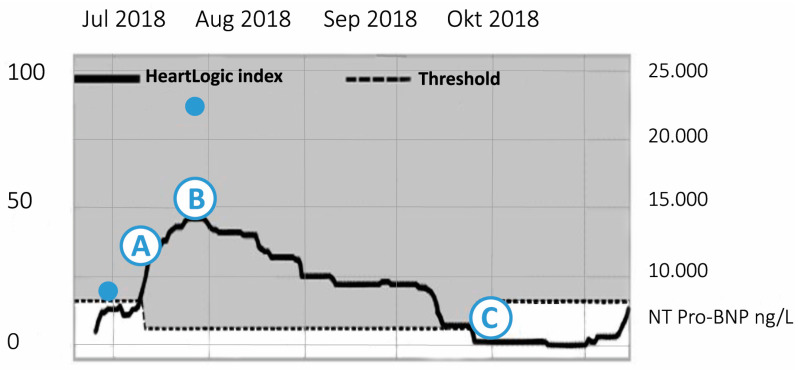
The course of HeartLogic index of patient described in Case 3. A (17 July): Phone contact. HeartLogic^TM^ index raises to 38. B (14 August): Phone contact. HeartLogic^TM^ index 50, NT-pro BNP (N-terminal B-type natriuretic peptide) 22.919 ng/L. C (26 October): Phone contact. Gradual decline of HeartLogic^TM^ until 0.

In April 2018, she developed a progressive cardiorenal syndrome with an admission because of decompensated heart failure. After recompensation with furosemide and dobutamine, an upgrade to a CRT-D device was performed because of intraventricular conduction disorder with a QRS duration of 140 ms. A few weeks later she was admitted to the surgical ward because of spontaneous bleeding from pseudoaneurysm of the arteria gastroduodenalis complicated by acute tubular necrosis and further renal deterioration. Although she retained residual diuresis, the renal function only partially recovered, and she was started on haemodialysis twice a week. During the outpatient clinic visit 1 month later, there were no signs of fluid overload and the HeartLogic^TM^ index was 5, Figure 7.

In July 2018, the HeartLogic^TM^ index rapidly increased to 38. The patient denied experiencing symptoms of fluid retention and was compliant with the lifestyle advice. According to the care path, a re-evaluation by phone followed 2 weeks later. At that time, the HeartLogic^TM^ index had increased to 50. She admitted having a higher salt intake and a slight increase in dyspnoea. She was in NYHA class III and physical examination revealed signs of fluid retention. The NT-proBNP had increased from 9500 ng/L to 22,000 ng/L. Despite an increase in the daily dosage of bumetanide to 15 mg/day, her fluid status could not be sufficiently optimised. Following a multidisciplinary consultation with the nephrologist, it was decided to extract extra fluid during each dialysis session. Subsequently, the HeartLogic^TM^ index decreased, ultimately to 1 and heart failure symptoms improved. This case illustrates that HeartLogic^TM^ can be used as a marker for fluid status in patients with complex haemodynamics, such as in cardiorenal syndrome.

## 5. Ongoing Studies

### 5.1. Literature Search of Ongoing Studies

A review of ongoing registered studies on the HeartLogic^TM^ algorithm was performed on 31 December 2020. To this aid, *ClinicalTrials.gov*, a database for clinical studies conducted around the world was used. The search was restricted to “HeartLogic” and “heart failure” patients. Two ongoing and one yet to be initiated study (n = 3) were extracted. Additionally, a recently conducted and not yet published study by Treskes et al. was retrieved from the Leiden University Medical Centre research registration. An overview of these four studies is provided in Table 2.

The validation of the HeartLogic^TM^ algorithm has by now been well established in the literature and it is clear that the ongoing studies are going to shift the focus from surrogate markers to clinical outcomes—including mortality, heart failure related hospitalisations and re-admissions.

### 5.2. The MANAGE-HF Study

The MANAGE-HF study is a randomised open label trial that has been enrolling patients since 2017. It will evaluate the clinical efficacy of CIED remote monitoring of heart failure patients with and without the HeartLogic^TM^ index in 2700 patients. The primary endpoint is all-cause mortality and secondary endpoint is heart failure related hospitalisations. The first results are expected in 2025.

### 5.3. The PREEMPT-HF Study

The PREEMPT-HF study is a prospective observational study that started enrolling patients in 2018. It will focus on association between HeartLogic^TM^ sensors and rehospitalisation within 30 days after an index heart-failure-related hospitalisation. The sensor value changes will be compared between the no readmission group and the readmission within 30 days group. It is expected that the HeartLogic^TM^ feature will be further enhanced based on the results of this study so that such alerts can lead to earlier intervention and reduction in heart failure admissions. Over 2100 patients have been included and patient recruitment was preliminary terminated in June 2020. Follow-up and data analysis are currently ongoing.

### 5.4. Other Ongoing Studies

A third study by Treskes et al. is a prospective non-blinded single-arm cohort study that has recently been completed and is currently under review for publication. It included 74 patients from three participating centres in the Netherlands, Belgium and Switzerland. A HeartLogic^TM^ pre-activation period of 365 days is compared with a 365 days post-activation period, primary endpoint consists of total number of hospitalisations for decompensated heart failure. Secondary endpoints are the number of patients hospitalised for heart failure, mean number of heart failure admissions per patient and mean length of hospital stay in days in the pre-activation period compared to the post-activation period.

The HeartLogic^TM^ France study is a prospective observational cohort study that was due to start enrolling patients in December 2020. Estimated enrolment consists of 300 patients. The study will focus on the annual heart failure hospitalisation rate as its primary endpoint. The secondary endpoints include: the annual cardiovascular and heart failure mortality rate, annual rate of unplanned hospitalisations due to ventricular and atrial arrythmias, annual rate of hospitalisation days related to heart failure, ventricular or atrial arrythmia, and patient quality of life. In addition, the study aims to perform weekly evaluation of the HeartLogic^TM^ index in all its subjects for the duration of a year

## 6. Future Perspectives

The HeartLogic^TM^ algorithm has extensively been validated and its association with, and role in, the prediction of impeding fluid retention several weeks prior to symptoms have been confirmed by numerous international studies. However, ongoing and future trials still have to clarify the impact of the HeartLogic^TM^ algorithm and timely HeartLogic^TM^ index guided intervention on hard clinical outcomes—including (reduction in) mortality, heart failure related hospitalisations and re-admissions. Furthermore, constraints on logistical implementation have to be resolved.

### 6.1. Clinical Implementation

In order to move forward, several practical aspects have to be considered. None of the above-mentioned studies evaluated the HeartLogic^TM^ index as a standalone technique to guide therapeutic actions. Patient care is not a “one size fits all”, and a HeartLogic^TM^ index guided clinical care path seems essential in this matter. Ideally, a standardised protocol should be at hand in which the roles of the individual care providers and the (therapeutic) consequences of a HeartLogic^TM^ alert are clearly defined. It still remains to be investigated whether mild symptoms or an alert by itself justifies therapeutic adjustments. In the MANAGE-HF trial [42], by design, patients in the intervention group receive a higher dose of diuretics in the case of a HeartLogic^TM^ alert, regardless of symptoms. However, due to the false alert rate and the potential side effects of high doses of diuretics (i.e., symptomatic hypotension, electrolyte disturbances, deterioration in kidney function), it can be argued that therapeutic action should only be undertaken in the case of concomitant symptoms. The manufacturer aims to enhance the HeartLogic^TM^ features based on the results of the PREEMPT study to optimise its role in guiding early therapeutic intervention in the prevention of heart failure readmissions [43].

Although the exact algorithm behind the cumulative HeartLogic^TM^ index is not released by Boston Scientific, it would be of interest to understand what the predictive values of the individual components are and correlate these to other remote monitoring strategies that are currently implemented in heart failure patients. In particular, implantable pulmonary artery pressure sensors, such as the CardioMEMS^TM^ sensor, enable daily measurement of left ventricular filling pressures, which have been shown to increase several weeks before a period of decompensation becomes clinically apparent [15,27]. Given the common hemodynamic sequelae evaluated by these techniques, it could be hypothesised that these sensors may have complimentary roles in the care of heart failure patients in the future.

### 6.2. Patient Selection

Future studies are expected to focus on the efficacy of the HeartLogic^TM^ features in specific patient groups—in particular, to guide care in those with advanced heart failure where conventional heart therapies and symptom management strategies are no longer effective, i.e., patients with a left ventricular assist device (LVAD) or those listed for a heart transplant or LVAD implantation; patients with (cardio-)renal failure requiring frequent fluid status optimisation by means of forced diuresis or dialysis and patients with pulmonary hypertension [44]. An important and often forgotten group of patients are those with heart failure with preserved ejection fraction (HFpEF). Approximately 50% of heart failure patients have HFpEF and, given the paucity of evidence-based treatments for HFpEF, it is not surprising that this patient group poses a high burden on healthcare resources and hospitalisations [45,46].

### 6.3. Logistics

To successfully implement a healthcare program, it is imperative to involve the different stakeholders. Regional collaborations with referring hospitals and general practitioners to be able to provide effective HeartLogic^TM^ and telemonitoring guided care “close to home” are essential. The insurance companies and the healthcare ministry are key players in healthcare financing and highlight the importance of not only focusing on the efficacy, but also evaluating the cost effectiveness of HeartLogic^TM^ guided care in various healthcare systems.

### 6.4. Limitations

This is a narrative review on the HeartLogic^TM^ algorithm. Several limitations should therefore be taken into account. We reviewed three electronic databases in search of literature and might have overlooked potentially relevant (ongoing) studies and therefore we acknowledge the possibility of skewed presentation. Other interesting developments in future sensors, including flexible electronics and wearable devices, are beyond the scope of this review and therefore not discussed in detail. To date, limited literature on the HeartLogic^TM^ algorithm is available; the majority of the studies are observational trials or case series with limited number of patients and relatively short duration of follow-up. In this review, a case series of three patients is shown to illustrate the implementation of the HeartLogic^TM^ algorithm in clinical practice. These cases provide no causal evidence and are illustrative by nature. The weight of each individual sensor in the algorithm of the HeartLogic^TM^ index is not released by the manufacturer. The potential impact of missing data collected by the CIED can therefore not be evaluated [47]. Data on hard clinical outcomes have not yet been published and are thus beyond the scope of this review.

## 7. Conclusions

The HeartLogic^TM^ algorithm has been successfully validated for early detection of fluid retention in an upcoming heart failure event. The results are promising, and several studies are currently focusing on hard clinical outcomes, such as (reduction in) mortality, heart-failure-related hospitalisations and re-admissions. A dedicated and well-defined clinical care path incorporating the HeartLogic^TM^ algorithm is required to maximise the benefit of “patient tailored care” and to limit the impact of clinically unexplained alerts. Further enhancement of the HeartLogic^TM^ algorithm and better understanding of the predictive values of alerts, addressing the logistical and financial aspects, are expected to contribute to a better integrated care service for patients with a CIED.

## Figures and Tables

**Figure 5:**
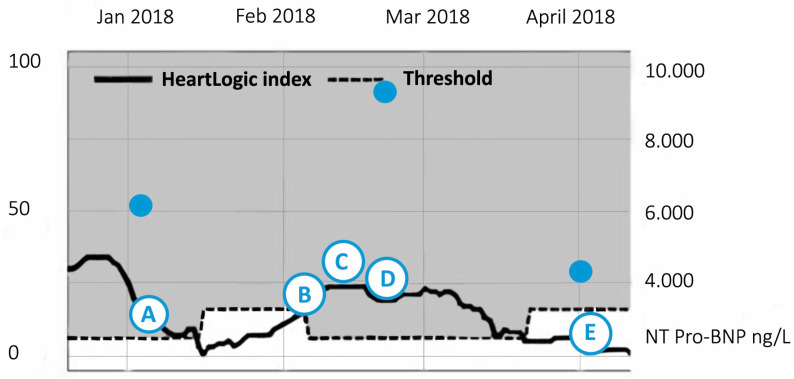
The course of HeartLogic^TM^ index of patient described in Case 1. A (3 January): Inclusion. HeartLogic^TM^ index 8, eGFR (estimated glomerular filtration rate) 44 mL/min/1.73 m^2^, NT pro-BNP (N-terminal B-type natriuretic peptide) 6474 ng/L. B (5 February): Phone contact. HeartLogic^TM^ index 17. C (15 February): Phone contact. HeartLogic^TM^ index 25. D (20 February): Out-patient visit. HeartLogic^TM^ index 22, eGFR 33 mL/min/1.73 m^2^, NT pro-BNP 9650 ng/L. E (2 April): out-patient visit. HeartLogic^TM^ index 0, eGFR 42 mL/min/1.73 m^2^, NT pro-BNP 4173 ng/L.

**Table 1:** Overview of the study characteristics of the 6 publications that fulfilled the literature search and selection criteria.

	Year	Study Design	Nr Pts	Mean Age in Years	Gender (Male in %)	Aetiology HF (ICM in %)	NYHA Class (I/II/III/IV in %)	LVEF (in %)	FU Time in Months	Primary Endpoint	Secondary Endpoint
**Boehmer et al. (Multi SENSE study)**	2017	Multicentre non-randomised trial	974	67	71	52	4/64/25/1	30	12	Predict sensitivity of 70% for heart failure event Alert 34 days prior to a HF event	Unexplained alert rate of 1.47 per patient year
**Gardner et al.**	2018	MultiSENSE study post hoc analysis	900	67	73	51	5/67/27/<1	30	12.9	IN alert state associated with 0.80 events per patient year vs. OUT of alert with an event rate of 0.08 events per patient year Event ratio IN/OUT 10.6.	50-fold risk of HF event when high NT-proBNP and IN alert vs. low NT-pro BNP and OUT alert state
**Capucci et al.**	2019	Retrospective case series	67	71	81	37	4/50/44/2	30	5 ± 3	1.0 alert per patient year (24 alerts in 16 patients) 2.0 12% of time spend in IN alert state	Unexplained alert rate of 0.41 per patient year
**Calò et al.**	2020	Multicentre prospective registry	104	71	73	40	2/44/51/3	29	-	S3 detects a restrictive filling pattern with 85% sensitivity and 82% specificity S1 detects LVEF < 35% with a 28% sensitivity and an 88% specificity	More impairment of systolic and diastolic function was associated with more frequent signs of functional limitation and congestion
**Santini et al.**	2020	Multicentre prospective registry	104	71	73	40	2/44/51/3	29	13	60% (60/100) of alerts were clinically meaningful 80% (48/60) of the clinical meaningful alerts were newly signalled by the algorithm 90% (43/48) of alerts triggered clinical action	Alert-based management strategy more efficient than a scheduled monthly remote (phone call) follow-up scheme
**Mitter et al.**	2020	Retrospective case series	38	60	76	-	18/63/18/0	32	3	A significant drop in activity may have resulted in less congestion	

Nr pts, number of patients; HF, heart failure; ICM, ischemic cardiomyopathy; NYHA: New York Heart Association functional class; LVEF, left ventricular ejection fraction; FU, follow-up; NT- proBNP, N-terminal pro B-type natriuretic peptide; S1, 1st heart sound; S3, 3rd heart sound.

**Table 2:** Overview of the study characteristics of the 4 ongoing studies.

	Start of Study	(Expected) Completion of Study	Study Design	Estimated Enrolment, (n)	Primary Endpoint	Secondary Endpoint
**MANAGE-HF**	2017	2025	Randomised open label trial	2700	All-cause mortality	Hospitalisation for heart failure
**PREEMPT-HF**	2018	2026	Prospective observational trial	2184 (current, recruitment terminated in June 2020)	Association of HeartLogic^TM^ sensors with 30-day heart failure re-admission	
**Treskes et al.**	2018	2020 (completed)	Multicentre non-blinded single-arm cohort	74	Total number of hospitalisations for decompensated heart failure, comparison pre- and post-activation	Number of patients hospitalised for heart failure Number of heart failure admissions per patient Duration of hospital admission
**HeartLogic^TM^ France study**	2020	2023	Prospective cohort study	300	Hospitalisation for heart failure	Annual cardiovascular mortality rate Annual mortality rate due to heart failure Annual rate of unplanned hospitalisations due to ventricular arrhythmia Annual rate of unplanned hospitalisations due to atrial arrhythmia Annual rate of hospitalisation due to heart failure, ventricular or atrial arrhythmia Patient quality of life using the Kansas City Cardiomyopathy Questionnaire Weekly evolution of the HeartLogic^TM^ index during a 12-month period

## Data Availability

Not applicable.
